# Capturing genetic variation in crop wild relatives: An evolutionary approach

**DOI:** 10.1111/eva.12626

**Published:** 2018-03-31

**Authors:** Paul A. Egan, Anne Muola, Johan A. Stenberg

**Affiliations:** ^1^ Department of Plant Protection Biology Swedish University of Agricultural Sciences Alnarp Sweden; ^2^ Department of Biology University of Turku Turku Finland

**Keywords:** adaptation, ecogeographic survey, flower frost tolerance, GIS, quantitative traits, rewilding, spatial genetic variation, wild strawberry

## Abstract

Crop wild relatives (CWRs) offer novel genetic resources for crop improvement. To assist in the urgent need to collect and conserve CWR germplasm, we advance here the concept of an “evolutionary” approach. Central to this approach is the predictive use of spatial proxies of evolutionary processes (natural selection, gene flow and genetic drift) to locate and capture genetic variation. As a means to help validate this concept, we screened wild‐collected genotypes of woodland strawberry (*Fragaria vesca*) in a common garden. A quantitative genetic approach was then used to test the ability of two such proxies—mesoclimatic variation (a proxy of natural selection) and landscape isolation and geographic distance between populations (proxies of gene flow potential)—to predict spatial genetic variation in three quantitative traits (plant size, early season flower number and flower frost tolerance). Our results indicated a significant but variable effect of mesoclimatic conditions in structuring genetic variation in the wild, in addition to other undetermined regional scale processes. As a proxy of gene flow potential, landscape isolation was also a likely determinant of observed patterns—as opposed to, and regardless of, geographic distance between populations. We conclude that harnessing proxies of adaptive and nonadaptive evolutionary processes could provide a robust and valuable means to identify genetic variation in CWRs. We thus advocate wider use and development of this approach amongst researchers, breeders and practitioners, to expedite the capture and in situ conservation of genetic resources provided by crop wild relatives.

## 1 INTRODUCTION

Greater utilization of genetic resources from crop progenitors and related wild species (i.e., crop wild relatives—CWRs) is urgently called for to increase agricultural productivity and sustainability (Dempewolf et al., 2014; Kell, Heywood, & Maxted, 2005; Kell et al., 2015; Lala, Amri, & Maxted, 2018; Vincent et al., 2013). As compared to their domesticates, CWRs typically harbour higher levels of genetic variation in key agronomically and horticulturally valuable traits (Fielder et al., 2015). Plant breeders exploit this wild diversity through a variety of traditional and modern genomic means, but generally seek to enhance or restore adaptive traits lost or degraded over the course of crop domestication. Commonly targeted genes and traits include those which underpin crop resistance to pests and disease, but have also included abiotic stress, yield and quality related traits (Dwivedi et al., 2008; Hajjar & Hodgkin, 2007; Maxted & Kell, 2009; Vincent et al., 2013). Such exploitation of CWRs in crop improvement programmes has grown steadily since the middle of the last century (Heywood, Casas, Ford‐Lloyd, Kell, & Maxted, 2007; Maxted & Kell, 2009). Accordingly, it has previously been estimated that about 30% of gains in crop yield worldwide—corresponding to an annual value of US $151 billion—could be attributed to the incorporated use of CWR germplasm (Pimentel et al., 1997). Furthermore, the frequency of release of cultivars containing CWR genes is set to further increase into the future (Dempewolf et al., 2017; Hajjar & Hodgkin, 2007; Lane & Jarvis, 2007), highlighting the renewed role for research in helping to meet this demand.

Despite their immense potential value, most CWRs remain insufficiently represented in genebanks (Castañeda‐Álvarez et al., 2016; Ford‐Lloyd et al., 2011) and are under increasing threat from anthropogenic‐related pressure (habitat loss, climate change, etc.) in the wild (Aguirre‐Gutierrez, van Treuren, Hoekstra, & van Hintum, 2017; Dempewolf et al., 2014; Jarvis, Lane, & Hijmans, 2008). In response to this situation, various national and global initiatives have been formulated which aim to systematically increase the field collection and in situ conservation of priority CWRs (Rubio Teso, Iriondo, Parra, & Torres, 2013; Singh et al., 2014; Fielder et al., 2015; Lala et al., 2018). Underpinning both these actions, however, is the need to first identify where high levels of genetic variation may be distributed intraspecifically in CWRs. To aid in this challenge, a technique known as “ecogeographic survey”—often implemented in geographic information systems (GIS)— has therefore been routinely employed (Gaston, Chown, & Evans, 2008; Maxted & Guarino, 2000). For this, known georeferenced occurrence records of CWRs are overlaid with various climatic or habitat datasets, which can permit identification of unique or priority populations for collection and/or conservation. Where genetic or evaluation data for specific traits are available, trait–climate correlations may also be derived, to extrapolate these relationships to areas where occurrence data are scarce or unavailable.

While many examples exist to date highlighting the successful application of ecogeographic survey, for example (Dulloo et al., 1999; Johnson & Vance‐Borland, 2016; Nkongolo & Nsapato, 2003), its utility in other cases has been limited (see (Endresen, 2010). This has been due, for instance, to the typically coarse resolution of bioclimatic data employed, which is generally not suited to the detection of local adaptation (Thormann, Reeves et al., 2016). Although issues of data coarseness potentially remain, refinements to this strategy, and the innovation of new tools and complementary approaches (e.g., ecogeographical land characterization (Parra‐Quijano, Iriondo, & Torres, 2012; Parra‐Quijano, Iriondo, & Torres, 2012), and predictive characterization techniques based on the Focused Identification of Germplasm Strategy (FIGS) (Thormann et al., 2014)), could therefore prove of large benefit.

Drawing on both established and novel concepts, we advance here an “evolutionary” approach to the identification and capture of genetic variation in CWRs. How adaptive and nonadaptive evolutionary processes act to shape genetic diversity is generally well understood in plants (Anderson et al., 2010; Etterson, Schneider, Gorden, & Weber, 2016; Linhart & Grant, 1996). However, despite these strong theoretical underpinnings, direct utilization of this knowledge in an applied, predictive sense for CWRs has to‐date been minimal (but see Berger, 2007; Warschefsky, Varma Penmetsa, Cook, & von Wettberg, 2014; Spooner, Ghislain, Simon, Jansky, & Gavrilenko, 2014; Johnson & Vance‐Borland, 2016; Thormann, Parra‐Quijano et al., 2016).

In practical terms, we consider how spatial proxies of evolutionary processes could be routinely developed for CWRs, to mirror three such processes: natural selection, genetic drift and gene flow. Already, FIGs‐based predictive characterization techniques are considered to target “adaptive” traits (Endresen, 2010; Maxted et al., 2016; Thormann, Parra‐Quijano et al., 2016) and in this sense harness natural selection as a driving force underlying trait diversification. However, practical use of other evolutionary processes (such as genetic drift and gene flow), and their potential integrated use alongside natural selection, remains unexplored. The development and use of a broadly encompassing evolutionary approach, as here proposed, thus offers an opportunity for evolutionary principles to better inform practice, and improve the efficiency by which genetic variation in CWRs can be identified and captured.

As a means to help validate the practical use of this approach, we collected and screened wild genotypes of woodland strawberry (*Fragaria vesca*) in a common garden, to assess quantitative genetic variation in three horticulturally important traits. The traits studied were plant size, early season flower number and flower frost tolerance. This information was then spatially related back to the original source populations, to test the ability of two proxies of evolutionary processes to predict genetic variation. Specifically, we tested whether genetic variation could be predicted (i) by local mesoclimatic variation (a proxy of natural selection)—as defined by common a priori*‐*selected climatic variables; (ii) by functional landscape isolation and geographic distance (proxies of gene flow potential); and (iii) by other undetermined spatial drivers (i.e., remaining significant variation not represented by the former processes). Based on these findings, we further elaborate on the concept of an evolutionary approach—including means of practical implementation, and the potential for integrated use with complementary strategies.

## 2 MATERIAL AND METHODS

### 2.1 Plant collection and cultivation

A total of 100 accessions of *Fragaria vesca* subsp. *vesca* were collected in the early spring of 2012 throughout an area spanning 8,209 km^2^ in Uppsala County, Sweden. Although this geographic area represents a relatively small expanse of the total distribution of *F. vesca*, this region is of particular significance climatically, as a transition zone between southerly continental and northerly boreal environmental zones (Metzger, Bunce, Jongman, Mücher, & Watkins, 2005). Accordingly, recent microsatellite marker analysis of seven of these collected accessions has confirmed high levels of neutral genetic differentiation between populations in this region (Hilmarsson et al., 2017).

In contrast to a strong emphasis on “within‐population” sampling in population genetic studies, ecological genetic (or genecology) studies typically seek to maximize sampling efforts across many diverse populations (Johnson & Vance‐Borland, 2016). Furthermore, given that genetic variation within *F. vesca* populations is often low (due to the predominance of asexual reproduction (Schulze, Rufener, Erhardt, & Stoll, 2012)), we collected single accessions from each population locality. A minimum of ca. 2 km was maintained between collection localities. The habitat of collection consisted of either forest or open, semi‐natural agricultural areas. At each locality, a runner was harvested from a randomly selected plant, from which clonal plantlets were propagated in a greenhouse. Upon reaching maturity, runners were then again collected and propagated. In late September 2013, juvenile plants (of the third generation, less than 3 month old) were transplanted into a common garden (located at 59.74°N, 17.68°E). As compared to agricultural trials, in which multisite and year experiments are routinely conducted to determine phenotypic stability of quantitative traits, single common garden experiments (as conducted here) are more typical of evolutionary studies (Moloney, Holzapfel, Tielbörger, Jeltsch, & Schurr, 2009) and nonetheless provide a sufficient means to assess genetic variation. Within the common garden, planting layout followed a randomized block design, in which one plant per genotype was grown in each of four blocks. The blocks were covered with a “blanket” (Lutrasil^®^ Pro 23 (23 g/m²)) immediately after planting, to increase winter survival. Plants were then allowed to establish for two growing seasons prior to trait measurements. A small amount of mortality randomly occurred during this time. We therefore limited the use of genotypes to those with full replication only (final *n* = 325 individuals, across 82 genotypes).

### 2.2 Trait measurements

Horticulturally important traits considered in this study included plant size, early flower number (as an indicator of early season yield) and flower tolerance to frost damage. Plant size appears to largely influence or covary with several other quantitative traits in woodland strawberry (personal obs.). We therefore confined our selection to the above three independent traits, which did not share more than moderate levels of phenotypic correlation (maximum *r* ^2^ across all pairwise correlations = .08). Plant size was quantified as the volume of a partial sphere (in dm^3^), derived from measurements of plant height and diameter. Total early season flower number per plant was recorded as the sum of flowers that had reached anthesis or postanthesis stage by 15 June 2015. We recorded flower frost tolerance as the inverse of the proportion of frost damaged flowers per plant. Frost damage is evident by a total blackening of the immature ovary, which subsequently fails to develop into a fruit. Trait values for plant size and early flower production were approximately normally distributed. Flower frost tolerance data were arcsine‐transformed to improve normality after first offsetting 0 and 1 values—which are problematic in angular transformations. This was achieved by replacing 0 values with (1/4n) and 1 with [1−(1/4n)], where *n* = the number of observations.

### 2.3 Genetic evaluation

For quantitative genetic analysis of clonally replicated genotypes, we used random effects (or variance component) models to analyse trait phenotypic variation across genotypes. As the error term of this model provided a measure of environmental (i.e., within‐genotype) variation, this model structure could hence be used to partition phenotypic variation into its genetic and environmental components (Hill, 2010; Nocetti et al., 2015). Accordingly, random‐effects models were fitted for each trait by restricted maximum likelihood (REML) and used to predict total genetic values for genotypes based on best linear unbiased predictors (BLUPs) (Piepho, Moehring, Melchinger, & Buechse, 2008). The models were fitted using the lmer function of the R package “lme4” (Bates, Mächler, Bolker, & Walker, 2014), implemented in R version 3.3.1. In addition to the random effect of genotype, a random block effect was also specified in each trait model, to control for within‐field variation. The resulting BLUP predictions for quantitative traits—thus corrected for block and environmental variation—were extracted from the model output. These BLUPs were utilized in subsequent analyses, either directly as genetic values, or for the purpose of calculating genetic distances between genotypes.

### 2.4 Climatic data

Climatic data were sourced from the Worldclim (v1.4) and Global Agro‐ecological Zones (GAEZ v3.0) bioclimatic datasets (Fischer et al., 2012; Hijmans, Cameron, Parra, Jones, & Jarvis, 2005). Before use, GAEZ raster data were resampled to a finer 1 km^2^ spatial resolution by bilinear interpolation (in ArcGIS 10.2.2), to match the resolution of Worldclim data. From a multitude of initial possibilities, we made an a priori selection of six commonly used climatic variables for use in this study. These included variables describing extremes in temperature (max. and min. T of the warmest and coldest months—in °C), precipitation (for the driest and wettest months—in mm) and two derived variables: length of the growing period (no. of days per year that T and soil moisture are conducive to plant growth) and the number of frost‐free days. As there was a strong negative correlation between these variables (*r* ^2^ = .55), we opted for use of the former, due to its greater predictive power in models.

### 2.5 Landscape metrics

Landscape analyses were based on a high‐resolution (25 × 25 m) raster land cover map for Sweden (“Svenska Marktäckedata” v1.1). According to this map, the focal region in which plant material was collected constituted a total of 56 unique land cover classes. To produce fewer more functionally relevant categories, we consolidated these into eight broad land class types (as listed in SI Table 1), using the “reclassify” tool in the spatial analyst extension of ArcGIS. Designation of these classes was guided by specific knowledge of habitat requirements for *F. vesca*, as well as land cover types not likely to support this species (Hancock & Bringhurst, 1978; Malinikova, Kukla, Kuklova, & Balazova, 2013; Roiloa & Retuerto, 2007; Schulze et al., 2012).

**Table 1:** Linear mixed model analysis of climatic effects on trait genetic variation in *Fragaria vesca*

Model	Plant size		Early flower no.		Flower frost tolerance^a^	
	*F*/*χ* ^2^ (df)	*R* ^2^	*F*/*χ* ^2^ (df)	*R* ^2^	*F*/*χ* ^2^ (df)	*R* ^2^
Fixed effects		.33		.17		.09
LGP	*F*_(1, 75)_ = **5.14** b		*F*_(1, 77)_ = 0.64		*F*_(1, 77)_ = 0.35	
Tmax	*F*_(1, 75)_ = **4.56** b		*F*_(1, 77)_ = 0.27		*F*_(1, 77)_ = **4.22** b	
Tmin	*F*_(1, 75)_ = 1.35		*F*_(1, 77)_ = 0.03		*F*_(1, 77)_ = 3.19	
Pwet	*F*_(1, 75)_ = 0.56		*F*_(1, 77)_ = 1.31		*F*_(1, 77)_ = 0.11	
Pdry	*F*_(1, 75)_ = 0.07		*F*_(1, 77)_ = **4.97** b		*F*_(1, 77)_ = 0.34	
LGP^b^Tmax	*F*_(1, 75)_ = **4.97** b		–		–	
Random effects						
Habitat	*χ* ^2^_(1)_ = 0.00		*χ* ^2^_(1)_ = 0.00		*χ* ^2^_(1)_ = 0.17	
Total model		.33		.17		.10

LGP,Length of the growing period; Tmax, Max. temp. of warmest month; Tmin, Min. temp. of coldest month; Pwet, precipitation of wettest month; Pdry, precipitation of driest month.

a Model validation indicated no significant difference against a null model (LR test: *L *=* *7.61, *p *=* *.18).

b *p *<* *.05 (in bold).

Habitat of provenance (either forest or open land) was included as a random effect.

See Methods for units of measurement

The reclassified map was imported into the program FragStats (version 4.2) for quantification of two landscape metrics: “similarity index” (SIMI) and “contrast‐weighted edge density” (CWED). Although strictly categorized as measures of landscape aggregation and contrast (McGarigal, 2014), respectively, we adapted use of these metrics (as detailed below) as two complementary measures of landscape isolation/connectivity. Each was parameterized as a functional metric—meaning that species‐specific information on *F. vesca* was used—and calculated over a circular radius of 10 km from the point of collection of genotypes. This distance is likely beyond the upper threshold of dispersal/gene flow for *F. vesca* within several generations, given the species' typical means of biotic dispersal (Muller‐Schneider, 1986; Willson, 1993). As inputs for the calculation of these metrics, similarity and edge contrast weights were first defined for all pairwise combinations of the eight land cover types (Table S1). These weights ranged from a value of 0 (no similarity/contrast) to a value of 1 (maximum similarity/contrast).

SIMI was thus calculated based on the similar weight, size and proximity of all deciduous and coniferous forest patches within a 10 km neighbourhood of each plant genotype point. As an inverse of isolation, high values of SIMI hence indicated the presence of large, proximate and ecologically suitable habitat patches in the surrounding landscape for *F. vesca*. CWED was calculated as the border (or edge) length between forests and other land class patches in the landscape, weighed by their relative structural contrast (e.g., structural contrast between forest and pasture was deemed high, and low between deciduous and mixed forest types—see Table S1). High values of CWED hence indicated a large amount of structurally contrasting forest edge surrounding a genotype point. In contrast to SIMI, which is positively weighed by the size of forest patches, CWED could capture the fact that many small patches of forest (with high edge density) could also serve to decrease isolation. Forest edges are particularly conducive to dispersal and gene flow for *F. vesca*, given its frequent association with disturbed, patchy environments.

### 2.6 Statistical analyses

We took a combined approach to analyses, in which plant traits were analysed as both a univariate and a single multivariate response. Univariate analyses were undertaken to detect whether and how traits responded differently to climate and functional isolation—whereas a multivariate approach permitted a wider focus on overall patterns. All analyses were conducted in R version 3.3.1 (R Core Team 2016).

#### 2.6.1 Mesoclimatic variation

Climatic effects on traits were analysed using individual linear mixed models (LMMs), to test for climatically structured genetic variation. Models were fitted by REML estimation in the “lme4” package—used in conjunction with the “lmerTest” package (Kuznetsova, Brockhoff, & Christensen, 2015) to estimate parameter significance. Climatic variables (as listed above) and their potential interactions were specified as fixed effects, and the habitat of each genotype's provenance (forest or open land) included as a random effect. Partial and total *R* ^2^ values from mixed models were generated using the package “MuMIn” (Bartoń, 2013). To check for possible multicollinearity amongst fixed effect variables, we monitored variance inflation factors (VIFs) using a custom function (developed by Austin F. Frank) to ensure that VIFs were observed as ca. 5 or under. Additional model validation was carried out, firstly through statistical and graphical assessment of standardized residuals for normality and homogeneity of variances, and secondly by testing that AIC (Akaike information criterion) of the mixed model was significantly lower than that of a null model containing only random effects.

#### 2.6.2 Landscape isolation

To assess the relative effects of landscape isolation and geographic distance on trait genetic values in *F. vesca*, statistical analyses were conducted using the MRM (“multiple regression on distance matrices”) function in the R package “vegan” (Oksanen et al., 2007). Based on pairwise differences between genotypes, Euclidean distance matrices were generated for the trait genetic values as response variables, and for the predictor variables of geographic distance, landscape similarity index and edge density. The test was performed using 10,000 permutations, and the results plotted (as raw distances) using the “added variable plot” function in the package “car” (Fox & Weisberg, 2011).

#### 2.6.3 Multiscale spatial variation

Spatial variation in trait genetic values was evaluated using a Moran's eigenvector approach, to test for patterns in genetic variation at multiple spatial scales. The analysis was applied in three steps, following the approach and notation outlined in Dray et al. (2012). In brief, utilizing “adespatial” (Dray et al., 2016) and associated R packages, analysis was conducted of: **Y**, a principle component analysis (PCA) of trait genetic values as a multivariate response; **F**, a multivariate multiple regression (redundancy analysis) of climatic and landscape explanatory variables on **Y**; and **R**, a partial residual analysis (a modified form of PCA) of the remaining variation, after partialling out the effects of climatic and landscape variables.

The resulting multivariate scores from **Y**,** F** and **R** were visually mapped and statistically analysed in a scalogram. Scalograms hence tested at which scale(s), if any, spatial patterns in genetic variation—and the effects of climatic, landscape and other undetermined processes—were evident. As inputs to produce the scalograms, the packages “spdep” and “adespatial” were first used to generate both a Gabriel graph (a type of connection network between spatial points—depicted in Figure 2), and a series of Moran's eigenvector map (MEM) variables, which simulated fine‐scale and broadscale spatial structures based on the Gabriel graph. This process generated a total of 81 MEM variables, which were divided into six groups in the scalogram, ordered from those which represented the broadest spatial scale (ca. 100 km) to the finest (ca. 2 km). The proportion of variance (*R* ^2^) explained by each spatial scale in the scalogram was then tested by means of a permutation procedure based on 999 repetitions.

#### 2.6.4 Variation partitioning

Variation partitioning was performed on trait genetic values (as a multivariate response—as in **Y**), to decompose the relative influences of climate, landscape isolation and spatial variation (at broadscale and fine‐scales). Prior to the analysis, we used a forward selection procedure to limit the number of MEM variables included to those which described significant variation only. Forward selection was implemented using “vegan” and employed the double stopping criterion of Blanchet, Legendre, and Borcard (2008) to reduce the chance of false positives and overestimation of explained variance. A total of six MEMs explaining 39.9% of the total variation were thus selected. These were divided into two groups corresponding to fine‐scale and broadscale variation—represented by two and four MEMs, respectively. Variation partitioning was then conducted using all four categories of variables (i.e., climatic, landscape and broadscale and fine‐scale spatial variation). For this, redundancy analysis (RDA) was used in “vegan” to compute adjusted *R* ^2^ values for each of the individual variables and their various combinations and to test the significance of the overall model.

Variation partitioning was performed on trait genetic values (as a multivariate response—as in **Y**), to decompose the relative influences of climate, landscape isolation and spatial variation (at broadscale and fine‐scales). Prior to the analysis, we used a forward selection procedure to limit the number of MEM variables included to those which described significant variation only. Forward selection was implemented using “vegan” and employed the double stopping criterion of Blanchet, Legendre, and Borcard (2008) to reduce the chance of false positives and overestimation of explained variance. A total of six MEMs explaining 39.9% of the total variation were thus selected. These were divided into two groups corresponding to fine‐scale and broadscale variation—represented by two and four MEMs, respectively. Variation partitioning was then conducted using all four categories of variables (i.e., climatic, landscape and broadscale and fine‐scale spatial variation). For this, redundancy analysis (RDA) was used in “vegan” to compute adjusted *R* ^2^ values for each of the individual variables and their various combinations and to test the significance of the overall model.

## 3 RESULTS

### 3.1 Mesoclimatic variation

Climatically structured genetic variation in *Fragaria vesca* was observed for plant size and early flower number, but not for flower frost tolerance (Table 1). The strength of climatic effects in structuring genetic variation in quantitative traits was thus variable, as indicated by the total model *R* ^2^ (which ranged from 0.10 to 0.33). Of the five bioclimatic variables examined, three of these (length of the growing period—LGP; maximum temperature of warmest month—Tmax; and precipitation of driest month—Pdry) successfully predicted trait genetic variation. For plant size, a significant negative interaction between LGP and Tmax was observed, indicating that LGP more strongly influenced plant size in areas where low values of Tmax occurred, and vice versa. High precipitation in the driest month positively impacted the number of early flowers produced. Outside of climatic influences, habitat of provenance (forest or open land) held very little influence on genetic variation (Table 1).

### 3.2 Landscape isolation

**Figure 1:**
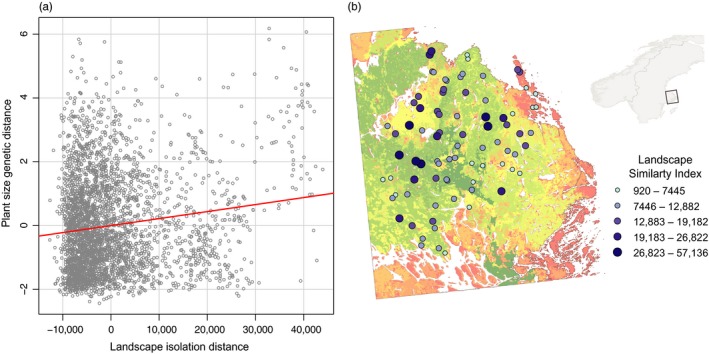
(a) Partial‐regression plot of the effect of landscape isolation (the inverse of landscape similarity index–SIMI) on genetic variation in plant size in *Fragaria vesca*. “Multiple regression on distance matrices” was performed using pairwise distances between all genotype points (as visualized by the *y*‐axis in a)—controlling for the effects of geographic distance and landscape edge density. (b) Plotted values of SIMI for each genotype point. Points are mapped against a regional surface map of landscape isolation, in which warmer colours indicate habitat patches with greater relative isolation from other similar patches in the region

The overall regression testing whether landscape functional isolation and geographic distance influenced trait genetic variation in *F. vesca* was significant for plant size (*F* = 30.8, *p *=* *.034), but not for early flower number (*F* = 5.2, *p *=* *.652) or flower frost tolerance (*F* = 11.2, *p *=* *.311). The partial‐regression coefficients of this model thus indicated a significant effect of landscape similarity index (SIMI) on plant size (*p *=* *.028, Figure 1.), whereas neither landscape edge density (CWED) nor geographic distance accounted for significant genetic variation in this trait.

### 3.3 Multiscale spatial variation

**Figure 2:**
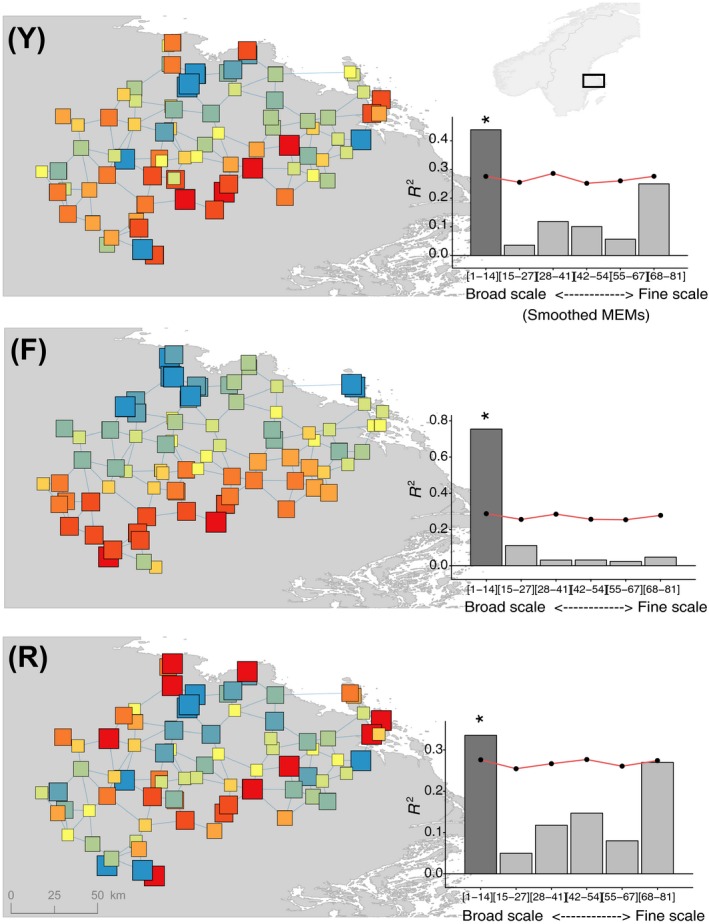
Multiscale analysis of trait genetic variation in *Fragaria vesca*. Plots illustrate the overall pattern of multivariate trait genetic variation (PCA analysis, **Y**), the pattern of environmental (climatic and landscape variables) influences on genetic variation (RDA analysis, **F**) and the remaining residual variation (**R**) after partialling out environmental effects. For each analysis, significant broadscale spatial variation is evident in the accompanying scalograms, in addition to notable but nonsignificant fine‐scale patterns. A red line indicates the .95 quantile for *R* ^2^ values obtained by permutation

A significant broadscale pattern of genetic variation was observed in multivariate analysis of quantitative traits (*R* ^2^ = .44, *p *≤* *.001), in addition to an important but nonsignificant fine‐scale component (Figure 2, top). Multivariate multiple regression (RDA) of the effect of climatic and environmental variables on trait genetic variation explained a significant proportion of variation (*F* = 2.6, *p *=* *.019, *R* ^2^ = .20). The RDA exhibited a single prominent axis (axis scores mapped in Figure 2, middle), correlating mainly with precipitation of the driest month (*r *=* *−.81), minimum temperature of the coldest month (*r *=* *−.66) and landscape edge density (*r *=* *−.64). The associated scalogram (Figure 2, middle) indicated a strong broadscale pattern of spatial variation (*R* ^2^ = .75, *p *≤* *.001)—consistent with the fact that most of the above explanatory variables varied at this scale. Partial residual analysis of the remaining unexplained variation (following the above two analyses) indicated notable fine‐scale and broadscale variation (Figure 2, bottom)—although only the latter was significant (*R* ^2^ = .34, *p *≤* *.008). Thus, other undetermined spatial drivers also appeared important in structuring genetic variation across the region of analysis.

### 3.4 Variation partitioning

**Figure 3:**
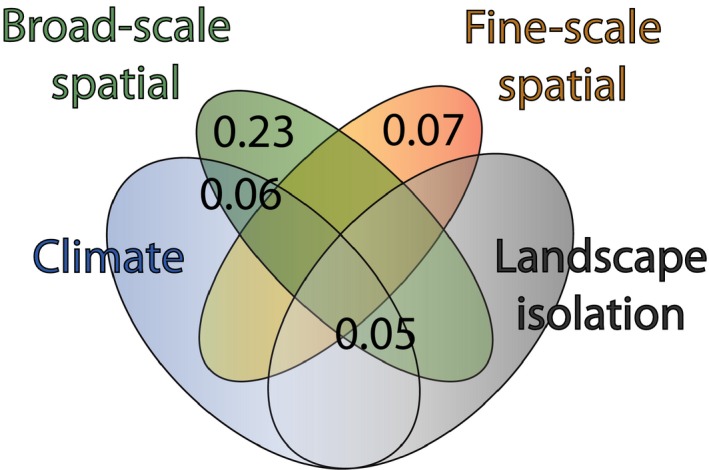
Partitioning of climatic, landscape and spatial (broadscale and fine‐scale) effects on trait genetic variation in *Fragaria vesca*. *R* ^2^ values are presented for significant fractions only. Only broadscale and fine‐scale spatial processes explained purely unique variation. Significant shared variation was, however, observed between broadscale and climatic and landscape fractions

Variation partitioning was performed to separate the relative influence of climate, landscape isolation and other undetermined fine‐scale and broadscale spatial processes on genetic variation. A considerable amount of variation (*R* ^2^ = .42) was as a whole explained by the model (RDA: *F* = 5.4, *p *<* *.001), of which variation partitioning (Figure 3) revealed the unique and shared contribution of the explanatory variables.

## 4 DISCUSSION

Our study demonstrates, in principle, how spatial proxies of evolutionary processes can be harnessed to identify and capture genetic variation in crop wild relatives. As proof of concept, we observed that such proxies were able to successfully predict spatial genetic variation in woodland strawberry, from local to regional scales. We further discuss these findings below, both in relation to woodland strawberry and in terms of the evolutionary approach as a whole.

### 4.1 Prediction of genetic variation in woodland strawberry

Climatic and landscape proxies of evolutionary processes (natural selection and gene flow potential) in general exhibited good predictive association with genetic variation in the studied *F. vesca* traits. We in addition note that the existence of broad genetic variation across the region of collection (e.g., as indicated by genetic distances in Figure 1a.) concurs with findings on neutral genetic variation in several of our collected accessions (Hilmarsson et al., 2017). Coupled with previous findings on the value of wild‐derived traits in this CWR (Muola et al., 2017), we surmise that the evolutionarily relevant proxies established here for *F. vesca* could be of value to future germplasm collection or preservation initiatives throughout this subspecies' wide Eurasian range.

In partitioning the relative predictive power of the employed evolutionary proxies, climate was deemed to be of marginally greater importance in structuring trait genetic variation. In univariate analyses of traits, the largest effect was observed for plant size. Aside from its positive association with yield in strawberry (Lacey, 1973), plant size is a key structural component of plant architecture—which amongst other qualities can influence the efficacy of biocontrol in cultivated systems (Cloyd & Sadof, 2000; Udayagiri & Welter, 2000). As a proxy for natural selection, mesoclimatic variation accounted for up to a third of genetic variation in plant size—due, prospectively, to local adaption across the region of study. In addition, the relative isolation of source populations, but not geographic distance between them also appeared important in structuring genetic variation in this trait. This relationship (depicted in Figure 1a) was significant while controlling for geographic distance—indicating, in other words, that potentially large genetic differences in plant size can be expected between populations which differ largely in their relative isolation, even where such populations are geographically proximate. This finding is hence consistent with our assumption that landscape isolation can act to constrain gene flow in *F. vesca* and thus serve as an effective proxy of this evolutionary process.

For early flower number, a positive association was observed with precipitation of the driest month, suggesting in addition the occurrence of climatically structured genetic variation. Early flower number is a good indicator of early season yield in strawberry. However, in many growing climates, flower frost damage presents a significant barrier to prolongation of the growing season (Neri, Baruzzi, Massetani, & Faedi, 2012; Sønsteby & Karhu, 2005). We observed little evidence of climatic control over flower frost tolerance, however. It is instead likely that adaptive variation in this trait may occur across larger geographic scales than as examined in this study.

### 4.2 An evolutionary approach

We define an evolutionary approach as any application or predictive use of evolutionary principles—spanning adaptive and nonadaptive processes—to guide the identification of genetic variation in CWRs. Given this broad definition, this approach does not necessarily differ in concept to other extant techniques which explicitly target “adaptive” variation (e.g., Thormann, Parra‐Quijano et al. (2016). Rather, a critical difference lies in its emphasis on holistic use of a broader range of evolutionary processes. Evolutionary processes are generally classified between those which serve to reduce genetic variation (natural selection, genetic drift), and those which act to increase it (mutation, recombination, gene flow). However, we here consider only three such processes—natural selection, genetic drift and gene flow—to operate in ways that may be spatially predictable and hence possess practical utility.

Implementation of an evolutionary approach may in practice revolve around three main considerations: (i) the extent of occurrence and relative importance of evolutionary processes in shaping spatial genetic variation in a given CWR; (ii) whether species‐specific proxies of these processes can be parameterized; and (iii) the most appropriate spatial scale(s) of focus. Table 2 provides examples of how such proxies may be devised for the three focal processes considered. In general, these feature the predicative use of abiotic and biotic variables as proxies of natural selection, alongside the demographic (size, age) and distributional (connectivity, disjunction) properties of populations and other spatial units as proxies of genetic drift and gene flow.

**Table 2:** Selected evolutionary processes—and their potential environmental correlates—which could be exploited to help capture genetic variation in crop wild relatives

Evolutionary process	Effect on genetic variation	Potential environmental correlates
Adaptive		
Natural selection	Reduction—Selectable genetic variation is decreased through removal of unfit variants (deleterious alleles), in favour of higher frequencies of better‐adapted phenotypes	Biotic and abiotic variation at local to regional scale
Nonadaptive		
Genetic drift	Reduction—Allele frequencies randomly increase or decrease from generation to generation, but through chance, may drift to fixation in a population's gene pool	Recent colonization (founder effects) Relative population size Large/long‐term geographic isolation or range disjunction
Gene flow	Increase—The addition of new alleles to a local gene pool via interpopulation migration can – up to a certain point—increase genetic diversity	Contact areas between distinct subranges or taxa (genetic admixture) Population connectivity (albeit risk of genetic homogenization under high connectivity)

Matching the search for adaptive trait variation to an appropriate spatial scale of focus is an issue of critical importance and has also been raised by previous authors (Endresen, 2010; Thormann, Reeves et al., 2016). Practical advice includes the need to employ fine‐scale climatic or environmental data in order to effectively target local adaptation and that selection pressures on adaptive traits (particularly biotic traits such as pest and pathogen resistance) can also occur equally strong over fine as for large geographic scales. The theoretical basis of this latter point has been well founded in the form of the “geographic mosaic theory of coevolution” (Gomulkiewicz et al., 2007; Muola et al., 2010).

The use of individual evolutionary proxies may in many cases be sufficient to adequately guide identification of genetic variation. However, simultaneous use of multiple proxies could also prove of particular benefit—given the added advantage that potential additive or interactive effects could be accounted for. For instance, where a strong abiotic gradient could serve as a useful proxy for natural selection in a target CWR, extensive gene flow could in part mask or confound this predictive ability, should the latter not be taken into account. In this sense, confounding evolutionary processes may offer one explanation for the weak predictive association observed in some past ecogeographic modelling studies (Endresen, 2010; Hijmans, Jacobs, Bamberg, & Spooner, 2003; Jansky, Simon, & Spooner, 2006). The net result of multiple interacting evolutionary processes may hence run contrary to expectations that selective pressure on adaptive traits should be apparent across wide ecogeographic gradients.

We in addition emphasize the potential conjoint utility of proxies of both adaptive and nonadaptive evolutionary processes—given the latter are so seldom considered in field studies of CWRs. In spite of this fact, we propose that targeting populations known or suspected to suffer from genetic drift or inbreeding depression (in which recessive alleles of certain genes may reach fixation in the gene pool) could nonetheless supplement the level of total genetic diversity available to breeders. Indeed, while this scenario can prove deleterious in wild populations, this need not necessarily be the case for purpose of plant breeding and cultivation.

In designing strategies for the collection and conservation of genetic variation explicitly guided by evolutionary principles, other targets of priority may include the following: 1.) areas of recent migration or contact between historically isolated populations or disjunct/minor subranges (e.g., hybridization zones between subspecific taxa), in which genetic admixture can promote the rapid evolution of unique genotypes (Rius & Darling, 2014; Salamone et al., 2013; Wagner, Ochocki, Crawford, Compagnoni, & Miller, 2017); 2.) relatively large‐sized populations, in which local adaptation is generally more common than in smaller ones (Leimu & Fischer, 2008); and 3.) the range margins of a CWR, where the direction, magnitude and tempo of natural selection and adaptation may differ largely from that of the range interior (Hill, Griffiths, & Thomas, 2011; Thomas, Bodsworth, Wilson, & Simmons, 2001).

Given the frequent absence of useful a priori information (landscape genetics, phylogeography, metapopulation dynamics, etc.) for many CWRs, good availability of spatial records and metadata (e.g., on occurrence, abundance, demography) housed in online databases and herbaria may otherwise prove valuable in implementing an evolutionary approach. For example, prioritized efforts have been made to systematically curate occurrence data on CWRs—an example of which is the Crop Wild Relative Global Occurrence Database (Castaneda‐Alvarez et al., 2014), which presents downloadable occurrence data gathered from hundreds of genebanks, herbaria and researchers. However, other population level data of potential evolutionary relevance (e.g., abundance, demography) remain unavailable for the majority of CWRs, even when national and/or taxon experts are consulted. As a possible means to surmount this problem, and in other cases where species‐specific parameterization of evolutionary proxies is not possible, approximation of relevant information (dispersal range, population size, connectivity criteria, etc.), or the use of generally informative variables (e.g., common primary climatic variables—as used in the present study), could offer a practical alternative.

## 5 CONCLUSIONS

Field‐based initiatives aimed at the collection and conservation of crop wild relatives have potentially much to gain from further explicit incorporation of evolutionary principles beyond the predominant focus on natural selection to date. The expanded evolutionary approach proposed in this study thus offers a valuable opportunity to realize this potential. We thereby envisage broad usage of this approach in practice (whether in stand‐alone applications, or conceptually embedded as part of other techniques), including amongst CWR researchers, field collection practitioners, conservationists and plant breeders. Placing new and improved tools into the hands of this community is vital towards meeting the urgent need to adapt crops and safeguard wild germplasm into the future.

Moving forward, exploring ways to further develop and integrate this expanded evolutionary approach with other established techniques (e.g., ecogeographic survey, ecogeographical land characterization maps, landscape resistance modelling, FIGs‐based predictive characterization) will prove especially valuable. While each shares some degree of conceptual overlap, such integration still undoubtedly offers potential for synergy. Spatial proxies developed for evolutionary process (e.g., as here for gene flow potential, Figure 1b) are particularly amenable to GIS‐based analysis. GIS can therefore offer an ideal platform in which to draw together and harmonize the diverse workflows of the above techniques. Integrated use of complementary techniques can in this sense offer strong potential for the future use and conservation of genetic resources provided by crop wild relatives.

## CONFLICT OF INTEREST

None declared.

## DATA ARCHIVING STATEMENT

Data for this study are available at the Dryad Digital Repository: https://doi.org/10.5061/dryad.g51kg15.

## Supporting information


** **
