# Supplementary material for: Capturing genetic variation in crop wild relatives: An evolutionary approach
Source: Evol Appl. 2018 Mar 31;11(8):1293–304. doi: 10.1111/eva.12626 (PMC6099816; doi:10.1111/eva.12626)
Supplement: Supplementary file 1 [file EVA-11-1293-s001.docx]

**Supporting Information**

**SI Table 1**. Similarity and edge contrast weights between each of the eight land cover classes which were used to calculate landscape metrics. For edge contrast weights, high structural contrast between land class types (e.g. between coniferous forest and cultivated land) were assigned a value of 1, whereas low edge contrast (e.g. between coniferous and deciduous forest) were assigned a value of 0. Corresponding land cover codes are listed for the SMD (Svenska Marktäckedata) and EU CORINE land cover maps.

| **Code** | **Land cover class** | **SMD code** | **CORINE**  **code** | **Similarity / Edge Contrast weights** | | | | | | | |
| --- | --- | --- | --- | --- | --- | --- | --- | --- | --- | --- | --- |
|  |  |  |  | 1 | 2 | 3 | 4 | 5 | 6 | 7 | 8 |
| 1 | Coniferous forest | 43-47 | 312 | - | 1 | 1 | 0 | 1 | 1 | 1 | 1 |
| 2 | Cultivated land | 30, 31 | 211, 222 | 0.15 | - | 0 | 1 | 0 | 0 | 0 | 0 |
| 3 | Inland waters | 80-82 | 511, 512 | 0 | 0 | - | 1 | 0 | 0 | 0 | 0 |
| 4 | Deciduous + Mixed forest | 40-42, 48-50, 53-55 | 311, 313, 324 | 0.66 | 0.17 | 0 | - | 1 | 1 | 1 | 1 |
| 5 | Urban | 1-14, 17-20 | 111, 112, 121-124, 131-133, 141, 142 | 0.05 | 0 | 0 | 0.05 | - | 0 | 0 | 0 |
| 6 | Pastures + natural grasslands | 32, 51 | 231, 321 | 0.27 | 0.44 | 0 | 0.83 | 0.16 | - | 0 | 0 |
| 7 | Marine waters | 83-86 | 521-523 | 0 | 0 | 0.5 | 0 | 0 | 0 | - | 0 |
| 8 | Other natural | 52, 58-61, 70-74 | 322, 331-334, 411, 412, 421 | 0.17 | 0.1 | 0.05 | 0 | 0.21 | 0.45 | 0 | - |
